# Attention-deficit/hyperactivity symptoms and personal strengths in adults

**DOI:** 10.1192/j.eurpsy.2024.790

**Published:** 2024-08-27

**Authors:** M. Miklósi, K. Vajsz, S. Oláh, V. Nagy, B. Szabó

**Affiliations:** ^1^Department of Clinical Psychology, Semmelweis University; ^2^Department of Developmental and Clinical Child Psychology, Eötvös Loránd University; ^3^Centre of Mental Health, Heim Pál National Pediatric Institute, Budapest, Hungary

## Abstract

**Introduction:**

Attention-deficit/hyperactivity disorder (ADHD) is one of the most prevalent chronic neuropsychiatric disorders in children and adolescents; however, it continues into adulthood in 4-77% of the cases. Due to executive dysfunction, adults with ADHD may have deficits in personal strengths, as well as difficulties utilizing existing strengths in challenging situations, which may add to the functional impairments associated with ADHD in adults.

**Objectives:**

Therefore, we aimed to explore the association between personal strengths and ADHD symptoms in a community sample of adults.

**Methods:**

Five hundred and twenty-eight adults (mean age = 21.41 years, *SD* = 2.29, range: 18 – 28, female: *N* = 488, 92.4%) filled out an online questionnaire after giving their informed consent. Personal strengths were assessed using Bernstein’s Strengths Scale (BSS) which measures sixteen positive attributes grouped into four higher-order factors: self-directedness (Identity, Self-reflection, Self-confidence, Self-assertion, Imagination/Creativity), self-regulation (Emotional balance, Resilience, Self-control, Self-care, Reality testing), connection (Empathy, Compassion, Humour, Responsibility), and transcendence (Gratitude and Wisdom). ADHD symptoms were measured by the screening version (Part A) of the Adult ADHD Self-Report Scale (ASRS).

**Results:**

Participants who were screened positive in ASRS (*N* = 247, 46.7%) scored lower in all but four BSS subscales than participants who were screened negative (*N* = 280, 53.3%). Effect sizes reached the medium level (Cohen’s d > .5) for Self-confidence, Self-care, Responsibility and Wisdom, and were small (Cohen’s d > .2) for Identity, Self-assertion, Imagination, Resilience, Reality-testing, Emotional balance, and Gratitude. However, no group differences were found in the Self-reflection, Empathy, Compassion, and Humour subscales of the BSS.

**Conclusions:**

Our results suggest that ADHD symptoms in adults may be associated with deficits in personal strengths; that is, adults with ADHD may have difficulties, especially in trusting in their abilities, qualities, and judgements, in taking care of their own emotional and physical well-being, in taking responsibilities and in being open to learning from their experiences. Addressing personal strengths in psychosocial interventions for adult ADHD may improve patients’ functioning.

This research has been supported by the National Research, Development, and Innovation Office, OTKA-PD-134849 and ÚNKP-22-2-I-ELTE-854 grants.

**Disclosure of Interest:**

None Declared

